# An Evasive Case of Gonococcal Endocarditis

**DOI:** 10.7759/cureus.44890

**Published:** 2023-09-08

**Authors:** William N Butler, Connor Stephenson, Benjamin D Young, Pranav Shah

**Affiliations:** 1 Internal Medicine, Medical University of South Carolina, Charleston, USA

**Keywords:** disseminated gonorrhea, mitral valve replacement, plasma microbial cell-free dna next generation sequencing, karius test, valvular endocarditis, gonococcal endocarditis

## Abstract

*Neisseria gonorrhoeae* is one of the most common sexually transmitted infections in the United States, and disseminated infection can lead to a variety of complications. This includes the less common, but potentially life-threatening complication of gonococcal endocarditis. The authors report a case of a formerly incarcerated middle-aged man with a three-day history of dyspnea on exertion, fever, headache, and productive cough with green sputum. He endorsed a several-week history of an untreated right molar infection but denied any history of genitourinary symptoms. Given concerns for heart failure, a transthoracic echocardiogram was obtained showing mitral regurgitation with a mass on the mitral valve leaflet, as well as a smaller aortic valve mass that was subsequently confirmed with a transesophageal echocardiogram. Initially, the patient was transferred from an outside hospital (OSH), and discrepancies were noted between the blood cultures obtained at the OSH and a private lab. Given that the patient was already started on antibiotics prior to transfer, a Karius assay was sent and returned positive for *N. gonorrhoeae.* He was started on empiric antibiotic coverage before ultimately undergoing mitral valve replacement with a mosaic valve. The patient completed six weeks of intravenous ceftriaxone with complete resolution of symptoms. This case demonstrates a rare incident of *N. gonorrhoeae* bacteremia without any common symptoms causing endocarditis and valvular destruction. Timely diagnosis, a multidisciplinary approach, and treatment of gonococcal endocarditis led to positive outcomes in this case.

## Introduction

In 2021, more than 710,000 cases of *Neisseria gonorrhoeae* (Gonorrhea) were reported in the United States, making it the second most common sexually transmitted infection after Chlamydia based on the Centers for Disease Control surveillance [1]. From a record low incidence in 2009 of 301,174, this is a 118% increase [1]. Subsequently, US-based public health services and international organizations have highlighted the importance of asymptomatic gonorrhea screenings in at-risk patients. When paired with the increase in antibiotic resistance demonstrated over the last few decades [2], the urgency to identify and treat gonococcal infections early, and appropriately, takes precedence.

As cases of gonorrhea rise, so does the inherent risk of more severe disseminated infections. Historically, gonorrhea progresses to disseminated gonococcal infection (DGI) in 0.5-3% of patients with untreated disease [3]. The pathogenesis of disseminated disease involves unregulated bacterial growth invading surrounding blood vessels, resulting in bacteremia and sporadic colonization throughout the body. Therefore, presentations of DGI can vary widely, from tenosynovitis and polyarthralgia to endocarditis and meningitis. Of these, endocarditis is a rare presentation, with it being a presenting sequelae in 1-2% of patients [4]. Here, we present a case of a 52-year-old male with disseminated gonococcal endocarditis.

## Case presentation

A 52-year-old male with a past medical history of hypertension, unspecified anemia, and polysubstance abuse presented to an outside hospital (OSH) complaining of three days of worsening dyspnea, fever, headache, and cough productive of green sputum. His social history was notable for polysubstance abuse, including cocaine and marijuana, although denied any history of intravenous (IV) drug use. He had been imprisoned for a total of 18 years and was released from incarceration 10 years prior to this presentation. He also reported a several-week history of a right upper molar infection with significant pain that he had not been seen for.

On presentation to the outside emergency department, he was noted to be febrile to 100.5°F, tachycardic to 106 bpm, and hypertensive to 155/108 mmHg. Notable labs included a leukocytosis of 15K/cumm, hemoglobin of 8.3gm/dL, sodium of 128mmol/L, creatinine of 2.3mg/dL, and B-type natriuretic peptide (BNP) of 635pg/mL. Physical exam was notable for a 4/6 systolic murmur heard at the cardiac apex and bilateral crackles heard best in the lung bases and during inspiration. A chest X-ray obtained at that time showed left greater than right opacities, trace pleural effusion, and pulmonary edema, and a computerized tomography chest with angiography showed no pulmonary embolism. In the setting of pulmonary edema and an elevation in BNP, a transthoracic echocardiogram (TTE) was obtained which demonstrated normal ejection fraction (60-65%), normal wall motion, mild tricuspid regurgitation, moderate to severe mitral regurgitation with a hyperechoic mass on the anterior mitral leaflet (1.2x1.8cm) with a resultant posteriorly directed mitral regurgitation jet, and a hyperechoic mass on the aortic valve (0.7x0.8cm). He was given 1L IV fluids, 40mg IV furosemide, and started on ceftriaxone and doxycycline before being transitioned to vancomycin and cefepime. The decision was made to transfer the patient to a tertiary care center with access to cardiothoracic (CT) surgery.

**Figure 1:**
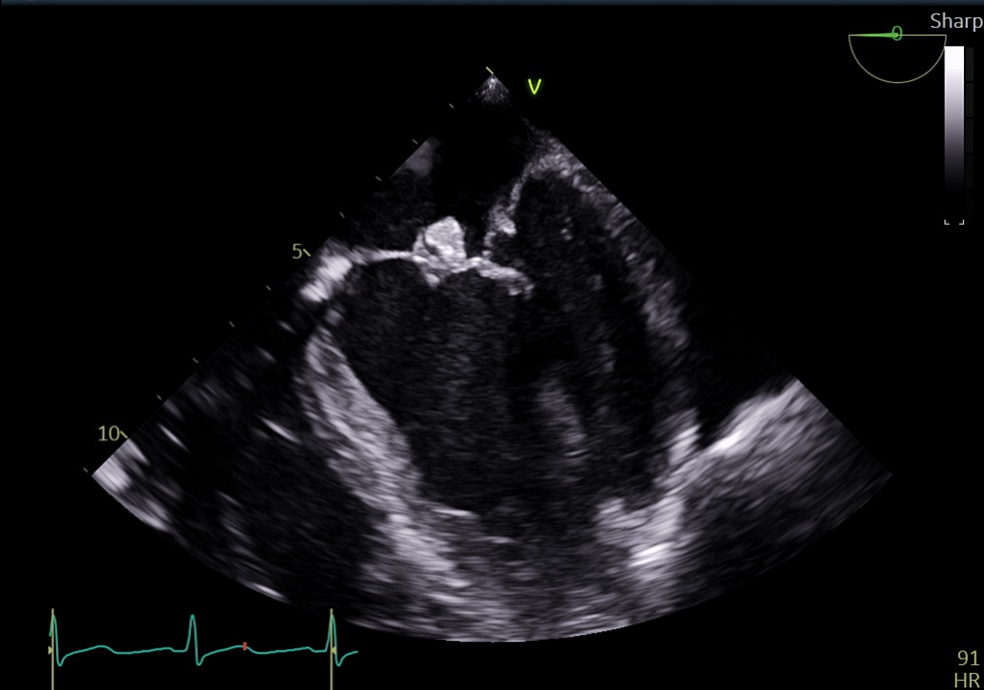
Midesophageal four-chamber view demonstrating vegetation measuring 1.1cm x 1.0cm on anterior mitral leaflet

**Figure 2:**
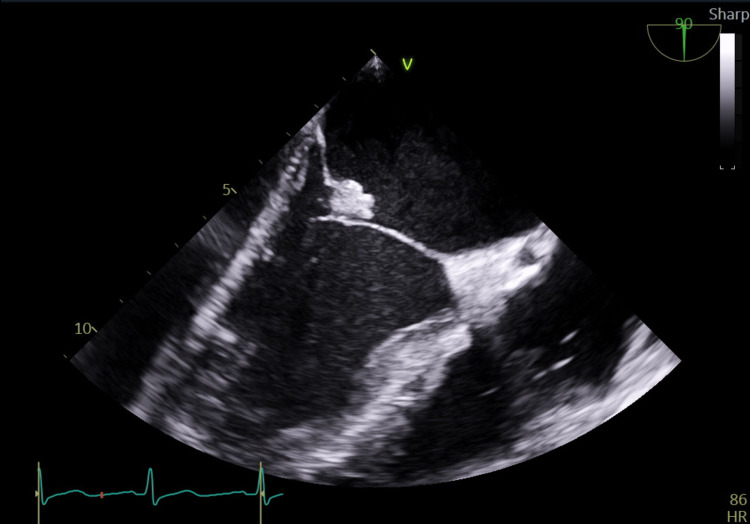
Midesophageal two-chamber view demonstrating anterior mitral leaflet vegetation and severe left atrial enlargement

After arrival at our tertiary care center, consults were made for CT surgery, cardiology, infectious disease, and oral maxillofacial surgery. He was continued on vancomycin (1750mg daily) and cefepime (1g every 12 hours), along with metronidazole (500mg every 8 hours) for oral anaerobe coverage. Further imaging was obtained including computed tomography angiography (CTA) of the head and neck, CTA transcatheter aortic valve replacement protocol, a repeat TTE, and a transesophageal echocardiogram (TEE) (Figure 1 and Figure 2). Imaging at our center confirmed the mitral valve vegetation but failed to demonstrate the aortic valve mass reported by the OSH. Diuresis was initiated with IV furosemide with a goal of 2-3L net negative.

At the time of arrival, blood cultures from the OSH were still pending as they had to be sent to a private lab, but the hospital lab reported 2 out of 2 Gram stains with gram-positive cocci in clusters and *Staphylococcus *species (not MRSA/MSSA) per polymerase chain reaction (PCR). However, the private lab ultimately isolated two out of two cultures growing *N. gonorrhoeae*. In speaking to the lab director the discrepancy was thought to most likely be due to an error with the Gram stain done at the OSH. Blood cultures obtained after arrival did not show any growth. Cefepime and metronidazole were discontinued, and ceftriaxone (2g every 12 hours) and doxycycline (100mg every 12 hours) were added for coverage of *N. gonorrhoeae* and *Chlamydia trachomatis*. Due to the discrepancy of these results, blood was sent for Karius testing, a cell-free DNA-based test, which confirmed *N. gonorrhea* with a presence of 663 molecules per microliter (MPM) (Ref: < 10MPM). Urine nucleic acid amplification testing (NAAT) was negative for both *N. gonorrhoeae* and *C. trachomatis*, and he denied any history of genitourinary symptoms. The patient denied any possible exposure to *N. gonorrhoeae* and denied any sexual contact outside of his wife.

Eighteen days after his initial presentation to the OSH, he was taken for mitral valve replacement (MVR) with a Mosaic valve by CT surgery due to the size of the vegetation and the severity of his mitral regurgitation. Cultures from the valve taken in the operating room (OR) demonstrated no growth of organisms. He recovered in the ICU and received resuscitation with subsequent diuresis. Post-op TTE demonstrated a prosthetic mitral valve in place with no evidence of mitral regurgitation. He was continued on IV ceftriaxone for the completion of six weeks of antibiotic therapy following MVR. At a six-week follow-up in the infectious disease clinic, the patient reported a complete resolution of symptoms.

## Discussion

While the incidence of gonococcal endocarditis has diminished greatly in the antibiotic era, rising rates of antibiotic resistance seen in *N. gonorrhoeae* [5], along with increasing rates of *N. gonorrhoeae* in the United States [1] could reasonably lead to an increase in the total number of disseminated infections and endocarditis. In the pre-antibiotic era, gonococcal endocarditis was an almost uniformly fatal disease [6] and thus increasing antibiotic resistance represents a concerning threat. Since the introduction of sulfonamides in the 1930s, *N. gonorrhoeae* has shown susceptibility to penicillin, tetracyclines, fluoroquinolones, and macrolides only to later develop resistance. Currently, the regimen of choice is dual therapy with an extended spectrum of cephalosporin (ceftriaxone) and azithromycin, however, cases of resistance to this regimen were first seen in 2018 [5]. Thus, while uncommon, *N. gonorrhoeae* is an important causative pathogen to include on the differential in any patient presenting with concern for infective endocarditis. A full sexual history should be obtained to identify any risk factors for *N. gonorrhoeae* infection.

According to one review, gonococcal endocarditis is primarily a disease of the young, with most cases occurring in patients aged 15-35 with no underlying valvular disease [6]. Almost all cases were associated with a new regurgitant murmur as well as signs of congestive heart failure. The most common indication for surgery in these patients is worsening symptoms of heart failure, as was the case with this patient. The most commonly affected valves are the aortic valve followed by the mitral valve, and 80% of cases have purely left-side involvement. Our patient with a new onset mitral regurgitation seen on echocardiography and appreciated on physical exam was consistent with these trends. Over half of all reviewed cases ultimately required surgical management of the affected valve, and there was an overall mortality of 19% in all cases.

*N. gonorrhoeae *finds success with infections primarily based on a variety of defense mechanisms. First, the bacteria utilize a type IV pili for a multitude of immune-evading features. Initially, it is used to attach itself to the epithelial cells of the host [7]. Once attached, these pili assist other bacteria with aggregation, further amplifying the initial infection. In addition to these physical attachments, gonococcal outer membrane proteins play a key role in the dissemination of systemic infections. PorBA1 and PorB1B, two outer membrane proteins expressed by *N. gonorrhoeae*, bind to complement-regulatory proteins C4bp and factor H [8], increasing their ability to evade complement immune components, thus increasing the ability of *N. gonorrhoeae* to disseminate systemically. For this reason, there is also a reported association between DGI and terminal complement deficiencies [9].

In this particular case, final diagnosis and targeted antibiotic therapy were delayed due to the difficulty in culturing the causative organism, *N. gonorrhoeae*. Initial reports from the OSH indicated the presence of gram-positive organisms on Gram stain which was inconsistent with the outside lab’s report of blood culture growth of *N. gonorrhoeae*. The reason for this discordance was unclear but was presumed to be a result of laboratory error. At the time of admission to our facility, cultures continued to be negative, notably after antibiotic therapy had already been initiated. In addition up to 50% of cases of DGI can have negative blood cultures [10]. Nonetheless, confirmatory testing may be obtained with the Karius blood test which has been demonstrated to be an effective technique to identify causative organisms in infective endocarditis when blood cultures are negative [11].

## Conclusions

Gonococcal endocarditis is a rare complication of *N. gonorrhoeae* infection that poses a risk of delayed treatment due to the high degree of suspicion needed to make the diagnosis. While uncommon, increasing antibiotic resistance of *N. gonorrhoeae* and increasing incidence could lead to an increase in the number of cases seen. Our case demonstrates the difficulty in making the diagnosis and the utility of Karius blood testing for confirmation. With proper identification of the causative pathogen, our patient was able to be successfully treated with no further symptoms after six weeks of targeted antibiotic therapy and valve replacement surgery.
